# Myelin status is associated with change in functional mobility following slope walking in people with multiple sclerosis

**DOI:** 10.1177/2055217318773540

**Published:** 2018-04-27

**Authors:** EM King, MJ Sabatier, M Hoque, TM Kesar, D Backus, MR Borich

**Affiliations:** Neuroscience Graduate Program, Emory University, USA; Division of Physical Therapy, Emory University School of Medicine, USA; Division of Physical Therapy, Emory University School of Medicine, USA; Shepherd Center, USA; Division of Physical Therapy, Emory University School of Medicine, USA

**Keywords:** Multiple sclerosis, myelin, myelin water fraction, magnetic resonance imaging, downslope walking, mobility

## Abstract

**Background:**

The level of myelin disruption in multiple sclerosis patients may impact the capacity for training-induced neuroplasticity and the magnitude of therapeutic response to rehabilitation interventions. Downslope walking has been shown to increase functional mobility in individuals with multiple sclerosis, but it is unclear if myelin status influences therapeutic response.

**Objective:**

The current study aimed to examine the relationship between baseline myelin status and change in functional mobility after a walking intervention.

**Methods:**

The Timed Up and Go test was used to measure functional mobility before and after completion of a repeated, six-session slope walking intervention in 16 participants with relapsing–remitting multiple sclerosis. Multi-component T_2_ relaxation imaging was used to index myelin water fraction of overall water content in brain tissue compartments.

**Results:**

Results demonstrated that the ratio of the myelin water fraction in lesion to normal-appearing white matter (myelin water fraction ratio) significantly predicted 31% of the variance in change in Timed Up and Go score after the downslope walking intervention, where less myelin disruption was associated with greater intervention response.

**Conclusions:**

Myelin water content fraction ratio may offer a neural biomarker of myelin to identify potential responders to interventions targeting functional impairments in multiple sclerosis.

## Introduction

Multiple sclerosis (MS) is a progressive neurologic disease that involves degeneration of the myelin sheath in the brain and spinal cord. Symptoms of MS, such as limb weakness, gait instability, and fatigue, lead to chronic disability and a decrease in functional mobility.^1,2^ Previous studies have shown that clinical responses to interventions in people with MS (pwMS) are widely heterogeneous, due to factors including disease duration, lesion volume, and level of disability.^3^ There is a need to better predict who will respond to a given treatment in order to tailor interventions to each individual.^4^ Evidence suggests that early therapeutic intervention is the most effective course of action.^4^ Better characterization of current disease status may guide development and personalization of interventions for pwMS.^5^

Downslope walking (DSW) on a treadmill has been identified as a potential therapeutic intervention utilizing repeated eccentric muscle contractions that forcibly lengthen the muscle and increase range of motion.^6^ DSW has been shown to decrease spinal excitability in healthy individuals, possibly due to an increase in descending cortical input.^7^ At a group level, DSW has been shown to improve functional mobility in a cohort of pwMS;^8^ however, individual variation in intervention response limits clinical translation and implementation. Currently, neural mechanisms underlying intervention response to DSW have not been systematically evaluated in pwMS. The neurobiology underlying the clinical response to DSW may inform the design and implementation of more effective rehabilitation interventions, such as DSW, for pwMS.

The primary pathophysiological feature of MS is myelin degradation due to immunopathology and neurodegeneration;^1^ thus, identifying a potential non-invasive marker of myelin status across disease course could provide important insights into the pathology of MS. Areas of demyelination, or ‘lesions,’ in pwMS can be identified in vivo as regions of abnormal signal intensity in conventional magnetic resonance imaging (MRI) scans.^9^ MRI-based measurements of tissue abnormality, or lesion volume, have previously been correlated with measures of physical and cognitive disability^10^ and have also been shown to be predictive of long-term disability.^11^ Longitudinal progression of T_2_ hyperintensities in white matter has been shown to be predictive of relapse rate and effectiveness of disease modifying therapies.^12^ Imaging markers have demonstrated evidence of structural plasticity in response to non-pharmacological rehabilitation interventions;^13^ however, little is known about the association between pre-intervention imaging markers of white matter status and response to rehabilitation-based interventions in pwMS.^5^ Quantitative metrics of lesion burden alone are unable to distinguish between different underlying pathological processes, such as inflammation, gliosis, and axonal injury.^14^ Therefore, there is a need to better characterize the effects of MS on specific tissue components of white matter.

Multi-component T_2_ relaxation imaging (MCRI) is an MRI approach used to evaluate water content in different brain tissue compartments.^15^ Myelin water fraction (MWF), the ratio of the water content trapped within the lipid bilayers of myelin to intra/extracellular water content, is an *in vivo* histopathologically-validated imaging marker of human brain myelin content obtained using MCRI.^16^ MWF has previously been shown to be decreased in white matter lesions compared to normal-appearing white matter (NAWM) in pwMS.^17^ Thus, the MWF can uniquely non-invasively index the degree of myelin disruption in the human brain to track disease progression and impact of myelin status on intervention response.

The current model of MS symptom development involves two factors: early inflammatory demyelination followed by neurodegeneration. The relationship between these two processes and their specific contributions to symptom development are not fully understood,^1^ and existing treatment options yield inconsistent results.^4^ Therefore, it is necessary to investigate the neural substrates underlying functional impairment and therapeutic response to rehabilitation interventions to inform the personalization of treatment plans in pwMS. The objective of the current study was to evaluate MWF, an in vivo marker of brain myelin status, as a potential predictor of the magnitude of change in functional mobility in response to a DSW intervention in pwMS.

## Methods

### Participants and study design

**Figure 1.**
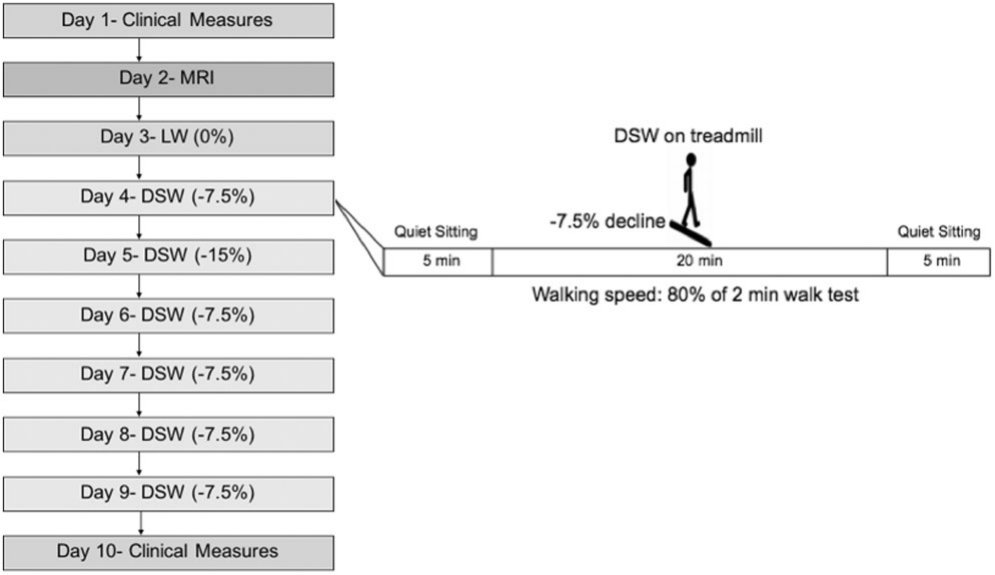
Study design. Left: Timeline of study design. Right: example of treadmill walking protocol during the first downslope walking (DSW) intervention visit. The same protocol was followed for all treadmill walking sessions (Days 3–9). Expanded Disability Status Scale (EDSS) and Timed Up and Go (TUG) test were administered on Day 1. Follow-up TUG test was performed on Day 10. MRI: magnetic resonance imaging. LW: level walking.

Sixteen participants diagnosed with relapsing–remitting MS were recruited through the Emory University Rehabilitation Hospital and Shepherd Center (Table 1). Exclusion criteria included: (a) a diagnosed MS relapse during the six months before enrollment; (b) history of cardiovascular disease; (c) history of epileptic seizures; (d) lower motor neuron disease; (e) unstable fracture of the lower limb or trunk; (f) inability to tolerate upright sitting for at least one hour; or (g) contraindications to MRI. All participants provided written informed consent in accordance with the Declaration of Helsinki. All study procedures were approved by the Institutional Review Board of Emory University and Shepherd Center. Participants completed a total of 10 experimental sessions, with a minimum of 24 h between consecutive sessions (mean inter-session period: 4.7 (±3.4) days) (Figure 1).

**Table 1.** Participant demographics.

Participant ID	Age (years)	Gender	EDSS	TUG day 1 (s)	TUG day 10 (s)	Lesion volume (mm^3^)
01	51	F	6.0	21.0	14.0	1024
02	39	F	2.5	13.5	10.0	5803
03	34	M	4.5	13.0	13.5	9741
04	54	F	1.0	10.0	8.5	4833
05	66	F	4.0	14.5	13.0	13,291
06	48	F	4.5	13.0	9.5	7231
07	68	F	3.5	10.0	10.0	7315
08	44	F	4.5	14.0	11.5	3791
09	22	F	0.0	7.0	7.0	2699
10	56	F	6.5	18.0	25.0	6391
11	53	F	1.0	13.5	10.0	11,941
12	53	F	4.0	8.0	7.0	11,904
13	34	F	5.5	17.5	17.5	6140
14	47	F	3.5	10.5	11.5	8442
15	51	F	4.5	12.0	11.0	9406
16	33	F	4.0	8.5	Not completed	6478
Average	47.1	15F,1M	4.0^a^	12.8	11.9	7276.9

EDSS: Expanded Disability Status Scale; TUG: Timed Up and Go Test.

^a^Median.

### Clinical measures

The Expanded Disability Status Scale (EDSS)^18^ was administered at Day 1 to index baseline level of disability. The two-minute walk test was performed to determine gait speed for subsequent treadmill walking sessions. The Timed Up and Go (TUG) test was performed on Day 1 and Day 10 to measure change in functional mobility^19^ following the DSW intervention.

### DSW intervention paradigm

Participants first completed a single session of level walking (Day 3), followed by six sessions of downslope treadmill walking (Days 4–9) (Sole Fitness F85 treadmill). The treadmill walking sessions occurred with a minimum of 24 h between consecutive sessions (Figure 1). Five out of six sessions were conducted with a –7.5% decline. An exploratory 15% decline was tested for the second session (Day 5) to evaluate participant tolerance to an increased slope as part of a separate study. Treadmill walking was performed for 20 min each day, and speed was set at 80% of the two-minute walk test speed. Rating of perceived exertion (RPE) and heart rate (HR) were measured every five minutes during treadmill walking. Resting HR was also measured five minutes before and five minutes after each treadmill walking session.

### Image acquisition

Magnetic resonance (MR) images were acquired (Day 2) at Emory University Center for Systems Imaging on a Siemens Magnetom TrioTim syngo MR scanner. The following scans were acquired: (a) 3D T_1_ turbo field echo (TFE) scan repetition time (TR) = 2300 ms, echo time (TE) = 2.89 ms, flip angle Θ = 8°, field of view (FOV) = 256 × 256 mm, 176 slices, 1 mm slice thickness, scan time = 9.83 min); (b) whole-cerebrum 32-echo three-dimensional gradient- and spin-echo (3D GRASE) for T_2_ measurement (TR = 1000 ms, echo times = 10, 20, 30, 40, …, 320 ms, 28 slices, 4 mm slice thickness, slice oversampling = 0.0%, in-plane voxel size = 1 × 1 mm, receiver bandwidth = 1250 Hz/Px, transverse orientation, acquisition time = 14.08 min); and (c) 3D Axial T_2_ fluid-attenuated inversion recovery (FLAIR) (TR/TE = 2600 ms/3.02 ms, flip angle = 8°, FOV = 256 × 232mm, 1 mm slice thickness, 160 slices). MRI methodology can be seen in the Supplementary Material.

### Image analysis

**Figure 2.**
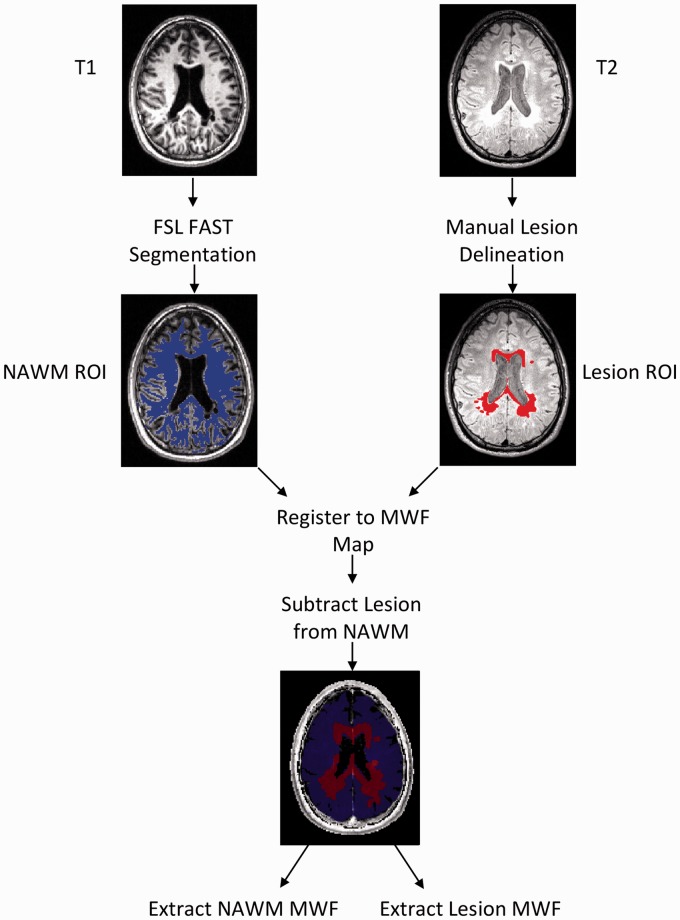
Magnetic resonance imaging (MRI) data processing pipeline. Normal-appearing white matter (NAWM) and lesion region of interest (ROI) masks were co-registered to the gradient- and spin-echo (GRASE) scan for each subject using FMRIB Software Library (FSL) FMRIB's Linear Registration Tool (FLIRT). Lesion masks were then subtracted from the NAWM mask to prevent overlap. Mean myelin water fraction (MWF) values were extracted for each ROI in each participant. FAST: FMRIB's Automated Segmentation Tool.

The T_2_ signal was separated into short (15–40 ms), medium (40–200 ms), and long (>1500 ms) components using a non-negative least squares (NNLS) approach^20^ with an in-house custom script (MATLAB R2017a, The MathWorks, Inc.). Voxel-based MWF maps (Figure 2) were generated by dividing the sum of the short T_2_ component amplitudes by the sum of the amplitudes for the total T2 signal (15–2000 ms) for each voxel.^21^ Higher MWF values represent higher myelin content.^16^

For each participant, two region of interest (ROI) masks were generated using the FMRIB Software Library v5.0^22^ (Figure 2):

1. NAWM ROI mask procedure: T_1_ images were skull stripped using the Brain Extraction Tool^23^ and then segmented into three categories, gray matter, white matter, and cerebrospinal fluid (CSF), using FMRIB's Automated Segmentation Tool.^24^ After visual inspection, the segmented white matter volume was used as the NAWM ROI mask.
2. Whole-brain lesion ROI mask procedure: white matter lesion masks were manually delineated using the T_2_ FLAIR image for all 16 participants by one rater and for a subset of five participants by a second rater to assess inter-rater reliability of manual ROI delineation. T_2_ hyperintensities were identified slice-wise to create the 3D lesion ROI mask.

**Figure 3.**
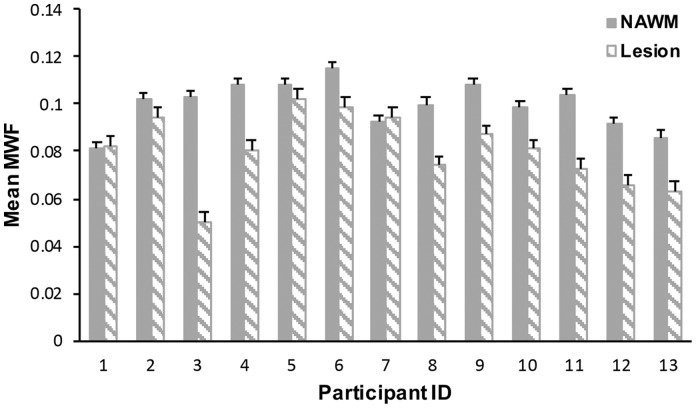
Mean normal-appearing white matter (NAWM) and lesion myelin water fraction (MWF) values across individuals. Mean lesion MWF was significantly lower than mean NAWM MWF (*p* < 0.001). Error bars represent standard deviation.

FMRIB's Linear Image Registration Tool was used to co-register the binary white matter mask and whole-brain lesion mask to the 3D GRASE image.^23^ An affine 12-parameter model with standard settings was used. The lesion mask was then subtracted from the NAWM mask to ensure no overlap between ROIs. The final, co-registered ROI mask was then overlaid on the short T_2_ component image to extract MWF values using an in-house Matlab script^25^ (Figure 2). A MWF ratio (lesion MWF/NAWM MWF) was also calculated to account for potential individual differences in NAWM MWF (Figure 3). MWF ratio values closer to one were indicative of less myelin disruption in lesioned regions.

### Statistical analysis

To assess inter-rater reliability of manually-drawn lesion masks, intraclass correlation coefficients (ICCs) were calculated for lesion volume and mean MWF values within each lesion ROI using a two-way random model. ICC values of above 0.75 were considered a priori to demonstrate excellent reliability.^25^

Shapiro-Wilk tests were performed on all dependent measures to assess normality. For those measures determined to significantly violate the assumptions of normality, non-parametric analyses would be performed. Paired-sample *t*-tests were planned to evaluate the difference in MWF between NAWM and lesion and, to evaluate the change in TUG test time after the DSW intervention (Day 10 vs Day 1). The difference in TUG test time (Day 10 TUG score minus Day 1 TUG score) was used as the primary outcome measure to index change in functional mobility after DSW.

Subsequently, due to limited sample size, an exploratory bivariate correlation analysis was conducted to evaluate relationships between change in TUG test score and neurobiological markers of interest (lesion volume, lesion MWF, NAWM MWF, and the MWF ratio). Results from the correlation analysis were used to identify the primary neurobiological predictor variable for a follow-up regression analysis to evaluate the association between the neurobiological marker identified and change in functional mobility after DSW. All statistical analyses were conducted using SPSS 24.0 (IBM, Armonk, New York, 2016). Significance level was set at *p* ≤ 0.05.

## Results

### Safety and intervention tolerance

One participant withdrew due to discomfort with treadmill walking procedures. No other adverse events were reported during walking. Across all walking sessions, average HR was 98.5 beats per min (range: 54–132, standard deviation (SD): 15.1), and RPE was 10.4 (range: 6–17, SD: 2.1).

### Normality of dependent measures

Shapiro-Wilk tests of normality indicated that lesion volume (statistic = 0.98, *p* = 0.98), TUGT test change (statistic = 0.92, *p* = 0.24), lesion MWF (statistic = 0.96, *p* = 0.70), NAWM MWF (statistic = 0.96, *p* = 0.77), and MWF ratio (statistic = 0.94, *p* = 0.47) were all normally distributed; thus, parametric analyses were performed.

### Difference in MWF between lesion and NAWM

Excellent inter-rater reliability was observed for lesion ROI volume (ICC = 0.99, *F*_4_ = 121.6) and mean MWF (ICC = 0.98, *F*_4_ = 59.4) using a manual lesion identification approach.

Mean (±standard error) lesion MWF (080±0.004) was significantly lower (*t*_15_ = 4.8, *p* < 0.001, difference = 19.4% (±1.5%)) than NAWM MWF (0.0999±0.003).

### Association between MWF and change in TUG test

At a group level, there was a mean reduction in TUG test time of 1.1 s after intervention (SD = 3.05), but the change was not significant (*t*_15_ = 1.4, *p* = 0.19, d = 0.36). Although group-level changes were not significant, nine out of 13 (69.2%) participants demonstrated a decrease in TUG test time (range: 1–7 s) after intervention.

**Figure 4.**
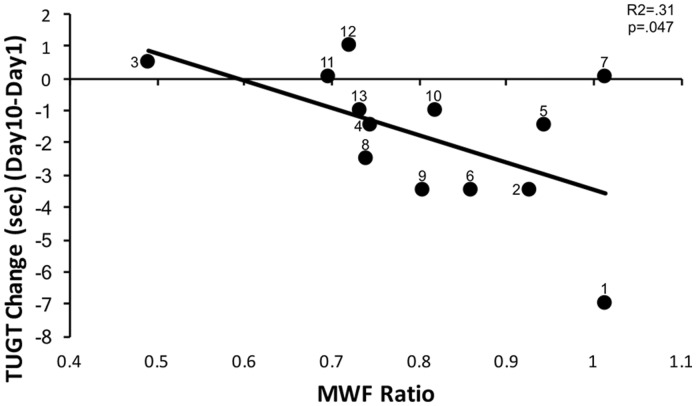
Myelin water fraction (MWF) ratio was associated with change in Timed Up and Go test (TUGT) score after downslope walking intervention (*R*^2^=0.31, *p*=0.047). Less myelin disruption in lesions, represented by higher MWF ratios, was associated with greater improvement (reduction in TUGT times) in functional mobility following the intervention. Participant ID numbers are provided beside each data point.

MWF ratio was significantly correlated with change in TUG test score (*r*=–0.56, *p* = 0.047). Lesion volume (*r* = 0.47, *p* = 0.11), lesion MWF (*r* = –0.45, *p* = 0.12) and NAWM MWF (*r* = 0.09, *p* = 0.76) were not significantly correlated with change in TUG test score. Thus, the MWF ratio was selected as the primary predictor variable for the regression analysis. Regression analysis revealed that MWF ratio predicted a significant amount of variance in change in TUG Test scores (*F*_1,12_ = 5.02, *p* = 0.047, *R*^2^ = 0.31) (Figure 4). Higher MWF ratios were associated with greater reductions in TUG test score after intervention completion.

## Discussion

The present findings report for the first time, to our knowledge, a predictive relationship between myelin status, indexed by a ratio of lesion MWF to NAWM MWF, and change in functional mobility following DSW in pwMS. The TUG test was used to assess functional mobility in the current study because the TUG provides greater task complexity than a simple walking task, includes sit-to-stand transition and turning, therefore more closely resembling the mobility demands of everyday life.^19^ Although the majority of participants demonstrated an improvement in time needed to complete the TUG test after the DSW, we did not observe a statistically significant group effect of DSW on TUG scores. However, this finding is consistent with previous studies that have shown that response to interventions in pwMS are widely heterogeneous, and there is a need to better predict who will respond to a given treatment in order to tailor treatment plans to an individual patient.^4^ Given that myelin disruption is a primary pathophysiological feature of MS,^1^ the significant association between myelin and intervention response could have the potential to inform personalization of rehabilitation interventions for pwMS. Neither clinical assessments nor total lesion volume were predictive of response to the intervention, further supporting the possible utility of quantifying human brain myelin status to predict intervention response.

Previous studies in pwMS have examined MWF in normal and abnormal (lesion) tissue separately;^17,26^ however, our results did not show a significant correlation between lesion MWF or NAWM MWF independently with response to intervention. Although not strongly associated with intervention response in our study, lesion MWF and NAWM MWF may be important markers of disease status in MS and may serve as useful predictors of intervention response either to DSW in larger study samples or to other behavioral interventions in MS. In the current study, using the MWF ratio provided information about the individual degree of myelin degradation in regions of overt pathology and related more significantly to change in functional mobility. Additionally, examining lesion volume alone does not provide information about the different pathological processes that contribute to symptomology.^14^ The degree of variability in NAWM MWF values observed in the current study (Figure 3) could be influenced by age or experience-dependent plasticity and could also be a result of microstructural damage not readily observable with conventional imaging in pwMS.^12^ Accounting for between-participant differences in NAWM myelin content provided an index of individual levels of myelin degradation in regions of overt pathology. Given the observed findings, MWF ratio may be more sensitive to inter-individual differences that relate more closely with clinical measures than MWF values extracted from areas of lesion alone.

Overall, our findings demonstrate that less myelin degradation was associated with positive response to DSW; consistent with previous studies showing that interventions are less effective as disease progression continues and myelin degradation increases. However, previous studies examined response to pharmacological interventions,^4^ whereas our intervention was rehabilitative in nature. Disease duration may be a factor in intervention response; however, it is difficult to determine onset of degeneration given demyelination may occur well before manifestation of behavioral symptoms^5^. MWF ratio may have potential as a neural biomarker to identify responders to intervention targeting functional impairments in MS, but future work is required to determine if a threshold MWF ratio for intervention response exists. It will also be important to determine how generalizable the predictive value of MWF is to other rehabilitative or pharmacological interventions.

Previous meta-analyses have shown that EDSS is related to potential response to pharmacological therapy.^4^ However, in the current study, EDSS score did not predict response to intervention. This may be due to small sample size or the unique characteristics of the downslope walking intervention that specifically targeted walking ability. Our findings may be consistent with the hypothesis that more complex motor tasks tend to require greater descending cortical control,^19^ therefore a marker of abnormal brain myelin content in pwMS may be more likely associated with TUG test performance than EDSS score given the task demands required. Lesion volume was not related to change in functional mobility, suggesting that the degree of myelin disruption within a lesion rather than the total volume is more strongly related to intervention response.

One limitation of the current study is the use of manual delineation of lesion ROIs. However, excellent inter-rater reliability was demonstrated and currently there is no universally accepted automated method for lesion identification in pwMS. Due to small sample size and heterogeneity of lesion location, we were unable to examine the influence of lesion location on our outcome measures. Another limitation is the lack of non-MS control group as well as a lack of control intervention group. However, the primary objective of the current study was not to determine therapeutic efficacy of DSW in pwMS, but to examine the relationship between myelin status and change in mobility associated with DSW. Additionally, the influence of spinal cord pathology on functional mobility^27^ was not examined in this study. We were unable to perform multiple regression analyses to investigate relationships between predictors due to sample size in the clinical cohort studied in this initial investigation.

## Conclusions

The key preliminary finding of the current study suggests that myelin status, as a potential brain-based biomarker of disease status, may be able to identify individuals most likely to respond positively to rehabilitation interventions (e.g. DSW) in pwMS. The utility of imaging myelin as a biomarker of therapeutic response in MS will need to be confirmed in larger study cohorts, and the generalizability of the current findings to other interventions requires investigation.

## Supplemental Material

Supplemental material for Myelin status is associated with change in functional mobility following slope walking in people with multiple sclerosis Click here for additional data file. Supplemental material for Myelin status is associated with change in functional mobility following slope walking in people with multiple sclerosis by EM King, MJ Sabatier, M Hoque, TM Kesar, D Backus and MR Borich in Multiple Sclerosis Journal – Experimental, Translational and Clinical
