# Supplementary material for: Myelin status is associated with change in functional mobility following slope walking in people with multiple sclerosis
Source: Mult Scler J Exp Transl Clin. 2018 Apr 27;4(2):2055217318773540. doi: 10.1177/2055217318773540 (PMC5954324; doi:10.1177/2055217318773540)
Supplement: Supplemental material for Myelin status is associated with change in functional mobility following slope walking in people with multiple sclerosis [file Supplemental_material.pdf]

MS Journal Appendix for MRI methodology

**Hardware**	
Field strength	3.0 T
Manufacturer	Siemens
Model	MAGNETOM TrioTim syngo
Coil type<br>(e.g. head, surface)	Head
Number of coil channels	16

**Acquisition sequence**		
Type<br>(e.g. FLAIR, DIR, DTI, fMRI)	3D T_1 MPRAGE	
Acquisition time	4.37 min	
Orientation	Sagittal	
Alignment<br>(e.g. anterior commissure/poster commissure line)	AC-PC	
Voxel size	1.0x1.0x1.0 mm	
TR	2600 ms	
TE	3.02 ms	
TI	800 ms	
Flip angle	8 deg	
NEX	1	
Field of view	256mm	
Matrix size	256 x 232	
Parallel imaging	Yes	No
If used, parallel imaging method:<br>(e.g. SENSE, GRAPPA)	GRAPPA	
Cardiac gating	Yes	No
If used, cardiac gating method:<br>(e.g. PPU or ECG)		
Contrast enhancement	Yes	No

Acquisition sequence	
If used, provide name of contrast agent, dose and timing of scan post-contrast administration	
Other parameters:	

Acquisition sequence		
Type<br>(e.g. FLAIR, DIR, DTI, fMRI)	whole-cerebrum 32-echo three-dimensional gradient- and spin-echo (3D GRASE)	
Acquisition time	14.08 min	
Orientation	Transversal	
Alignment<br>(e.g. anterior commissure/poster commissure line)	AC-PC	
Voxel size	1.5x1.5x4.0 mm	
TR	1000 ms	
TE	10, 20, 30, 40, …, 320ms	
TI		
Flip angle	90	
NEX	1	
Field of view	240 mm	
Matrix size	160 x 160	
Parallel imaging	Yes	No
If used, parallel imaging method:<br>(e.g. SENSE, GRAPPA)		
Cardiac gating	Yes	No
If used, cardiac gating method:<br>(e.g. PPU or ECG)		
Contrast enhancement	Yes	No

Acquisition sequence	
If used, provide name of contrast agent, dose and timing of scan post-contrast administration	
Other parameters:	

Acquisition sequence	
Type<br>(e.g. FLAIR, DIR, DTI, fMRI)	T_2 FLAIR
Acquisition time	6:24
Orientation	Transverse
Alignment<br>(e.g. anterior commissure/poster commissure line)	AC-PC
Voxel size	1.0x1.0x1.0 mm
TR	4500 ms
TE	366 ms
TI	1800 ms
Flip angle	120 deg
NEX	2
Field of view	250 mm
Matrix size	512 x 512
Parallel imaging	Yes No
If used, parallel imaging method:<br>(e.g. SENSE, GRAPPA)	GRAPPA

Acquisition sequence	
Cardiac gating	Yes No
If used, cardiac gating method:<br>(e.g. PPU or ECG)	
Contrast enhancement	Yes No
If used, provide name of contrast agent, dose and timing of scan post-contrast administration	
Other parameters:	

Image analysis methods and outputs	
Lesions	
Type<br>(e.g. Gd-enhancing, T2-hyperintense, T1-hypointense)	T2-hyperintense
Analysis method	Manual lesion drawing
Analysis software	FSL
Output measure<br>(e.g. count or volume [ml])	Volume (mL)
Tissue volumes	
Type<br>(e.g. whole brain, grey matter, white matter, spinal cord)	
Analysis method	
Analysis software	
Output measure<br>(e.g. absolute tissue volume in ml, tissue volume as a fraction of intracranial volume, percentage change in tissue volumes)	
Tissue measures (e.g. MTR, DTI, T1-RT, T2-RT, T2*, T2’, ^{1}H-MRS, perfusion, Na)	
Type<br>(e.g. whole brain, grey matter, white matter, spinal cord, normal-appearing grey matter or white matter)	Normal and abnormal white mater
Analysis method	Multi-component T2 relaxation with non-negative least squares (NNLS)
Analysis software	Custom Matlab scripts
Output measure	Myelin water fraction (MWF)
Other MRI measures (e.g. functional MRI)	
Type<br>(e.g. whole brain, grey matter, white matter, spinal cord, normal-appearing grey matter or white matter)	
Analysis method	
Analysis software	
Output measure	

Other analysis details:
